# Expression levels and activities of energy-yielding ATPases in the oligohaline neritid snail *Theodoxus fluviatilis* under changing environmental salinities

**DOI:** 10.1242/bio.059190

**Published:** 2022-02-11

**Authors:** Jan Knobloch, Christian Müller, Jan-Peter Hildebrandt

**Affiliations:** Animal Physiology and Biochemistry, Zoological Institute and Museum, University of Greifswald, Felix Hausdorff-Strasse 1, D–17489 Greifswald, Germany

**Keywords:** Osmoregulation, Snail, *Theodoxus*, Ion pumps, Changes in environmental salinity

## Abstract

The aquatic gastropod *Theodoxus fluviatilis* occurs in Europe and adjacent areas of Asia. The snail species has formed two genetically closely related subgroups, the freshwater ecotype (FW) and the brackish water ecotype (BW). Other than individuals of the FW ecotype, those of the BW ecotype survive in salinities of up to 28‰. Coastal aquatic ecosystems may be affected by climate change due to salinization. Thus, we investigated how the two *Theodoxus* ecotypes adjust to changes in environmental salinity, focusing on the question whether Na^+^/K^+^-ATPase or V-ATPase are regulated on the transcriptional, the translational or at the activity level under changing external salinities. Animals were gradually adjusted to extreme salinities in containers under long-day conditions and constant temperature. Whole body RNA- or protein extracts were prepared. Semi-quantitative PCR- and western blot-analyses did not reveal major changes in transcript or protein abundances for the two transporters under low or high salinity conditions. No significant changes in ATPase activities in whole body extracts of animals adjusted to high or low salinity conditions were detected. We conclude that constitutive expression of ATPases is sufficient to support osmotic and ion regulation in this species under changing salinities given the high level of tolerance with respect to changes in body fluid volume.

## Abstract (precis)

**Summary:** *Theodoxus fluviatilis* snails are mild hyperregulators that express basic levels of transport ATPases that are not regulated by external osmolality. Animals avoid body volume changes using organic osmolytes.

## INTRODUCTION

Ion-transporting ATPases are present in any animal cell and maintain ion gradients across plasma membranes. The activity of the widely expressed Na^+^/K^+^-ATPase, which pumps sodium ions from the cytosol to the extracellular space and, at the same time, accumulates potassium ions intracellularly (Horisberger, 2004; Bejček et al., 2021), accounts for up to 85% of the total metabolic rate of animals even in water-dwelling osmo-conforming species (Leong and Manahan, 1999) that do not maintain strong osmotic gradients between the environmental medium and the internal body fluids. Moreover, many types of epithelial cells express proton-pumping V-ATPases, which, besides being involved in the acidification of intracellular vesicles, reside in the plasma membrane and expel H^+^-ions from the cytosol to the extracellular space (Nelson and Harvey, 1999; Wieczorek et al., 1999). The resulting ion gradients across the plasma membrane are the basis of the electrical membrane potential and provide the driving forces for secondary transmembrane transport processes of other ions, sugars, amino acids, and nutrients in every cell in the body (Friedman, 2008).

In aquatic invertebrates, at least portions of the body surface (epidermis, gills, intersegmental membranes, etc.) are permeable to water. This is in part due to the presence of aquaporin water channels in cell membranes (Campbell et al., 2008) that may provide transcellular water permeability or to water permeability of tight junctions (Beyenbach, 2003), which control the tightness of the paracellular pathway. Both help to equilibrate the osmotic concentrations of different compartments within the animal's body by compensatory water fluxes or allow water transport along osmotic gradients between the animal's body and the environment. Thus, animals maintaining their internal body fluids at osmotic levels higher than that of the external medium (hyper-regulators) show an influx of water through the body surface. Volume homeostasis is maintained by urine production via excretory organs, which, in turn, results in steady loss of valuable osmolytes, mainly ions, from the body. Ion loss has to be compensated by active transport of ions from the dilute medium into the body through the integument which is energized by Na^+^/K^+^- or V-ATPases (Treherne, 1987; Lee et al., 2011). On the other hand, animals that are transferred in external salinities that exceed those of their body fluids suffer from volume loss through the integument. Volume homeostasis in such animals is usually maintained by drinking, which, in turn, enriches the body fluids with ions that have to be selectively pumped out of the body by transport ATPases (Takei, 2000).

Different species of gastropods inhabit terrestrial, marine, brackish water, or freshwater habitats. The aquatic species may be subdivided in truly marine species, freshwater species and oligohaline species, the latter occurring in brackish water of changing salinities (Deaton, 2009). While marine species and true freshwater species are generally osmoconformers (the internal osmolality is always close to the environmental osmolality), the oligohaline species are partial regulators that hyperregulate at low environmental salinities and are osmoconformers at higher environmental salinities. Despite these physiological differences the structures of the renal systems seem to be very similar in gastropods and specifically in species within the superfamily of Neritoidea (Little, 1972). Generally, hemolymph is filtered through the wall of the heart into the pericardium by a hydrostatic pressure gradient. The pericardial fluid passes to the kidney through the reno-pericardial canal. During passage through the more or less sculptured lumen of the glandular portion of the renal tubule system the fluid may be modified by secretion and reabsorption to form the final urine that is released to the mantle cavity through the uropore. Analyses of micropuncture samples taken from the visceral blood sinus, the pericardium, the anterior and posterior parts of the glandular portion of the kidney, and the urinary bladder revealed that purely marine species regulated their ion content to some degree but did not osmoregulate at all. Freshwater species and those living in brackish water, however, were able to reabsorb ions from the primary urine flowing through the glandular portion of the kidney by active transport through the epithelial cells. In consequence, these animals were able to excrete urine that was hypoosmotic with respect to the hemolymph. There are no studies of other tissues that may potentially have ion- or osmoregulating capabilities (epithelial cells of the integument, mantle cavity, ctenidium or the gastrointestinal system).

An example of such a freshwater/brackish water neritoid gastropod is *Theodoxus fluviatilis*, which modestly hyperregulates its hemolymph (approximately 100 mOsmol/kg; Neumann, 1960; Little, 1972) at environmental osmolalities below 80 mOsmol/kg H_2_O, but behaves as an osmoconformer at higher environmental concentrations (Symanowski and Hildebrandt, 2010). Snails of this species can be subdivided into two different ecotypes according to their habitats, freshwater lakes and streams (FW animals) or brackish water of the Baltic Sea (BW animals) (Neumann, 1960; Zettler et al., 2004), and several physiological parameters like the different abilities of animals from these groups to survive changing salinities (Symanowski and Hildebrandt, 2010; Wiesenthal et al., 2018). Furthermore, individuals of the different ecotypes have different modes of accumulating organic osmolytes under hyperosmotic stress (Wiesenthal et al., 2019).

In an attempt to understand the regulatory capabilities of snails of the FW- and BW-ecotypes, we compared expression levels and activities of transport ATPases, potentially relevant for survival under changing external salinities. We used transcriptomic data of this snail species (NCBI, accession number: SRR15300633) to derive specific primers for amplification of cDNAs of the alpha-subunit of the Na^+^/K^+^-ATPase or the A subunit of the V-ATPase, respectively, and monitored transcript levels by semi-quantitative RT-PCR in animals maintained in their original medium (freshwater, salinity of 0.5‰ or brackish water, salinity 8.0‰ or transferred in a stepwise manner to higher salinities (FW ecotype to 18‰, BW ecotype to 28‰) or to lower salinities (only BW ecotype to 0.5‰). These salinities were selected as they are challenging but not deadly for the animals (Wiesenthal et al., 2018). Using protein extracts of animals that had been treated accordingly, we measured abundances of transport ATPase subunits by semi-quantitative western blotting and determined specific ATPase activities. Our results indicated that both Na^+^/K^+^-ATPase as well as V-ATPase homologs are expressed in *Theodoxus fluviatilis*. Subtle changes in transcript levels, however, were not matched by alterations in protein abundance or specific ATPase activities. V-ATPase activities were at the detection limit at all conditions indicating that V-ATPase may not be important for ion homeostasis in these animals. The ion- and volume balances in these animals seem to depend on the basal activity of the Na^+^/K^+^-ATPase.

## RESULTS

**Fig. 1.**
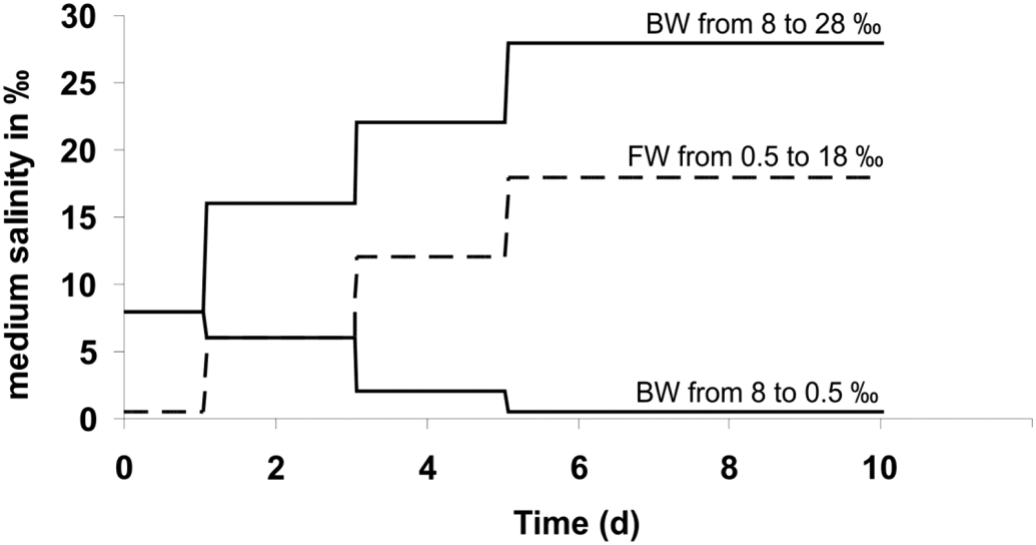
**Transfer regimes used to acclimate snails to media with different salinities.** Individuals of the FW ecotype were initially maintained in freshwater (salinity 0.5‰). Individuals of the BW ecotype were initially kept in brackish water with a salinity of 8‰. These salinities corresponded to those of the original habitats of the animals. The animals were then transferred in a stepwise manner over 4 days to their final salinities. The FW animals were transferred to a maximum salinity of 18‰. The BW animals were either brought to a final salinity of 28‰ or acclimated to 0.5‰. Animals were maintained at these salinities for another 5 days to allow for adjustments to reach new steady states.

The stability of specific mRNAs and their steady-state levels in cells are important factors in the control of gene expression and determine the translation rates of the respective proteins (Horvathova et al., 2017). Assuming that there is a correlation of transcript levels with levels of housekeeping protein expression (Schwanhäusser et al., 2013) also in our snails we expected to find higher expression levels of mRNAs and translation products of transport ATPases in animals under hypoosmotic stress (external medium<80 mOsmol/kg H_2_O) than those in their original medium due to the need for active ion absorption through the integument. Likewise, animals under hyperosmotic stress (external medium>80 mOsmol/kg H_2_O in FW snails or >230 mOsmol/kg H_2_O in BW snails) were expected to have higher expression levels of mRNAs as well as proteins for the transport ATPases than those in their original medium due to the need for excretion of surplus ions from the body fluids. The experimental regimes of acclimating animals to media with different salinities are shown in Fig. 1.

**Fig. 2.**
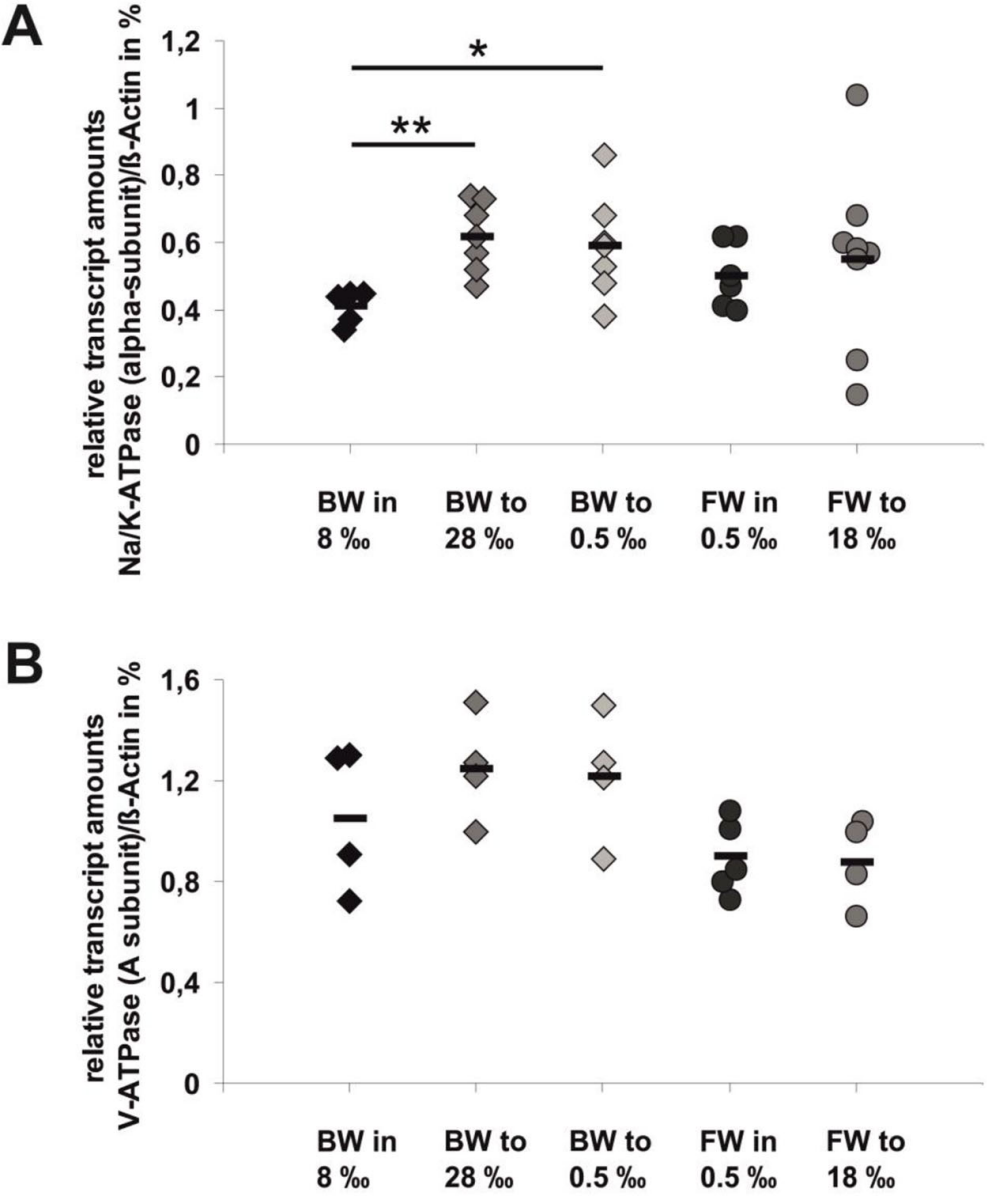
**Relative amounts of transcripts of transport ATPase subunits in whole body extracts of individuals of *T. fluviatilis* in their original medium or upon acclimation to changes in external salinities.** (A) Transcript amounts of the Na^+^/K^+^-ATPase alpha-subunit in individuals of the FW ecotype or the BW ecotype. (B) Transcript amounts of the V-ATPase A-subunit in individuals of the FW ecotype or the BW ecotype. Transcript amounts were normalized to the respective transcript amounts of beta-actin. The data shown represent individual measurements in each group (A: *n*=5–7, each; B: *n*=4–5, each) and the respective means (horizontal bars). Significant changes in transcript abundances compared with the values of animals in their original media are indicated: *P≤0.05; **P≤=0.01.

The transcript levels for the alpha-subunit of the Na^+^/K^+^-ATPase (Fig. 2A) in BW animals were indeed significantly higher (factors approximately 1.5x) in animals transferred to media with high (28‰) or low (0.5‰) salinities compared with the controls in their original medium (8‰). However, there was no effect on the transcript levels in FW animals transferred from their original medium (0.5‰) to a higher salinity (18‰). The transcript levels of the A-subunit of the V-ATPase (Fig. 2B) did not show any significant changes upon transfers of BW animals to high or low medium salinities or exposing FW animals to a high medium salinity, respectively.

Holoenzymes of transport ATPases are only released from the endoplasmic reticulum and send to their final locations in cells when the subunit composition is correct (McDonough et al., 1990; Tokhtaeva et al., 2009; Hou et al., 2020). Free subunits or incorrectly assembled molecules are rapidly degraded. Thus, the total amount of holoenzyme present in a given tissue can be judged from the analysis of individual subunits.

**Fig. 3.**
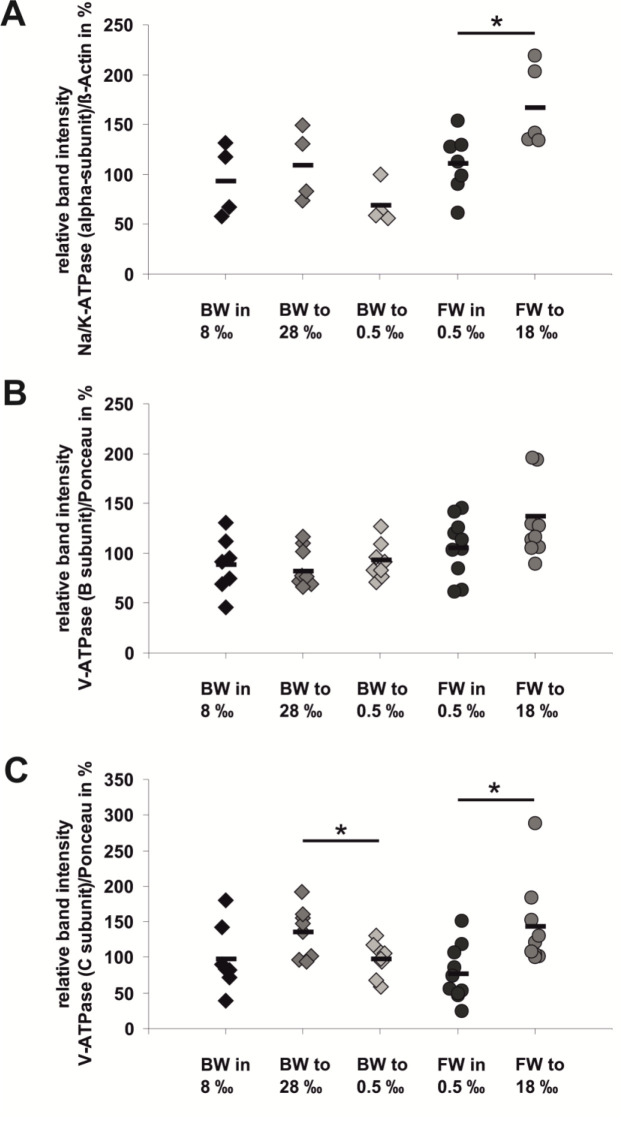
**Relative amounts of ATPase subunit proteins in whole body extracts of individuals of *T. fluviatilis* in their original medium or upon acclimation to changes in external salinities.** (A) Protein amounts of the Na^+^/K^+^-ATPase alpha-subunit in individuals of the FW ecotype or the BW ecotype. (B,C) Protein amounts of the V-ATPase B-subunit/C-subunit in individuals of the FW ecotype or the BW ecotype. Protein amounts were normalized to the respective amounts of beta-actin (A) or to a stable band on the Ponceau-stained blot (B,C). The data shown represent individual measurements in each group (A: *n*=4–7, each; B,C: *n*=8–10, each) and the respective means (horizontal bars). Significant changes in transcript abundances compared with the values of animals in their original media are indicated: *P≤0.05.

The protein contents for the alpha-subunit of the Na^+^/K^+^-ATPase (Fig. 3A) were not different in BW animals that had been acclimated to high (28‰) or low (0.5‰) salinity compared with those in their original medium (8‰). However, the alpha-subunit of the Na^+^/K^+^-ATPase was significantly more abundant (factor approximately 1.5x) in FW animals that had been transferred to 18‰ compared with FW animals that had been left in water with the original salinity of 0.5‰. The B subunit of the V-ATPase V_1_ complex did not show any significant changes in its abundance due to transfer of BW or FW animals from their original media in water with high or low salinities (Fig. 3B). The C subunit of the V_1_ complex of the V-ATPase, however, showed significant increases in its level (factors of 1.4 or 1.9, respectively) after animals had been transferred from their original media (BW 8‰ or FW 0.5‰, respectively) to high salinities (BW 28‰ or FW 18‰, respectively) (Fig. 3C). No effect on the expression level of the C subunit was detected after BW animals had been transferred to the low salinity medium (0.5‰).

**Fig. 4.**
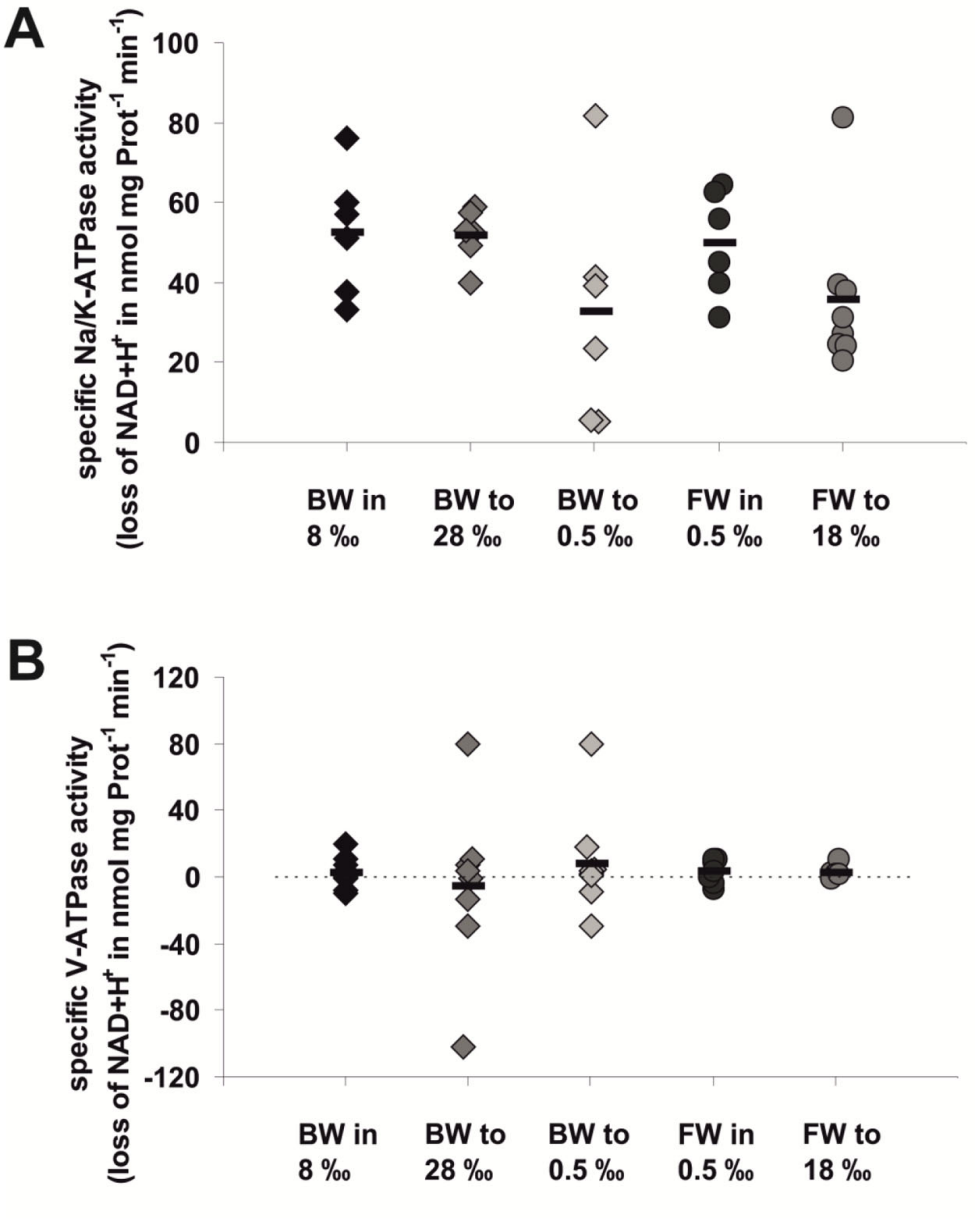
**Specific activities of Na^+^/K^+^-ATPase (A) or V-ATPase (B) in whole body protein preparations of individuals of *T. fluviatilis* in their original medium or upon acclimation to changes in external salinities.** Specific activities were determined by blocking the activity of Na^+^/K^+^-ATPase using 6 mmol/l ouabain or blocking the activity of V-ATPase by adding 1 µmol/l bafilomycin A1 to the assays. The data shown represent individual measurements in each group (*n*=8–9, each) and the respective means (horizontal bars).

Specific activities of transport ATPases were measured in protein preparations of snails that had been exposed to their original salinities (BW 8‰ or FW 0.5‰, respectively) or that had been acclimated to low (0.5‰) or high salinities (BW 28‰ or FW 18‰), respectively. Total ATPase activities (measured as the rate of decline in the original NADH content of the assay) were determined in parallel assays to those in which 6 mmol/l ouabain or 1 µmol/l bafilomycin A1, respectively, had been added to inhibit Na^+^/K^+^-ATPase- or V-ATPase-activities. The differences in the slopes of these curves were considered the specific activities of these transport ATPases. As shown in Fig. 4, no significant differences in specific activities of Na^+^/K^+^-ATPase (A) or V-ATPase (B) could be detected between protein preparations obtained from animals in their original media or those that had been acclimated to high or low salinity conditions. The Na^+^/K^+^-ATPase activities were between 33 and 53 nmol NADH per mg protein and minute and were in the same order of magnitude that had been measured in other invertebrates (Caruso-Neves et al., 1998; Leong and Manahan, 1997). The activity of the V-ATPase was basically at the baseline indicating that the animals did not use their V-ATPase for any transport activities associated with ion transport under conditions of changing salinities.

In an attempt to obtain information on the tissue-specific expression of the Na^+^/K^+^-ATPase alpha-subunit in *Theodoxus fluviatilis*, we tried to use the monoclonal a5 antibody (dilutions of 1:150 or 1:50) with an HRP-detection system in immune-histochemical assays on tissue sections of *Theodoxus* (cryosections as well as paraffine sections). Unfortunately, we failed to see any specific signals. We concluded that this antibody is not suitable for detecting the protein in tissue sections of gastropods.

## DISCUSSION

Phenotypic plasticity is a common phenomenon of functional adjustments of animals to environmental change, which is often associated with altered protein expression in specific cells or entire organs. In such cases, environmental changes have to be sensed by an organism and, upon proper information processing, casted into an appropriate response like changes in transcription rates of genes encoding relevant proteins (Evans, 2010). Moreover, stability or translation rates of mRNAs may be altered to change the levels of relevant proteins in the cells. Protein levels are, in turn, also affected by regulation of protein degradation. If protein degradation or translation rates are affected by environmental stress, regulation of gene expression may be required to maintain constant protein levels. Likewise, highly labile transcripts may require constantly high transcription rates to maintain sufficiently high protein levels. In other cases, the ultimate product of gene transcription may be regulatory RNA itself (e.g. antisense RNA). Therefore, although gene and protein expression are tightly coupled in some cases, changes in gene expression that do not accompany changes at the protein level may still reflect important changes to cellular function. In addition, post-translational modifications or changes in the subcellular localization of existing proteins add to the complexity of regulation of proteins in the cellular context. In effect, changes in transcription rates of certain genes may not always be directly related to changes in expression levels or biological activities of proteins (Gracey and Cossins, 2003; Logan and Buckley, 2015).

Ion transporting ATPases are generally considered to be important housekeeping proteins for animal cells because they provide the energy for all secondary transport processes of ions, nutrients, intermediate metabolites or waste products. Their functions are essential for maintaining homeostatic conditions in the animals and allow proper regulatory responses to environmental challenges (Wuytack, 2009).

In such cases of important housekeeping proteins, changes in gene transcription should have an impact on protein abundance. There are many examples of oligo- or euryhaline vertebrates or invertebrates that adjust protein expression and protein activity levels in transporting epithelia like integument or gills when experiencing salinity stress (Chung and Lin, 2006; Patrick et al., 2006; Tang et al., 2009; Faleiros et al., 2010; Reilly et al., 2011; Jonusaite et al., 2011; Tiburcy et al., 2013). While regulation of expression levels or transport activities of ion transporting ATPases may be expected in animals whose internal ion and osmotic concentrations are maintained at different levels from those of the external medium (regulators), it is interesting that there are reports in the literature about regulatory responses in expression levels of ion transporting ATPases in osmoconforming species like molluscs under salinity stress (Lin et al., 2016; Jia and Liu, 2018).

*Theodoxus fluviatilis* is a neritid snail that inhabits lakes and streams (freshwater) in Europe, but also the shores of the Baltic Sea where it lives in brackish water up to a salinity of 28‰. As there are no proven cases of hybrid populations in estuaries, it seems that the freshwater snails and the brackish water snails are fairly well isolated from each other (Zettler et al., 2004) despite their similarities in shell, operculum and radula features (Zettler, 2008) and in molecular markers (Bunje and Lindberg, 2007). Physiological investigations revealed that snails from both groups have a broad salinity tolerance range (oligohaline species) but are somewhat different in salinity tolerance. Brackish water animals and freshwater animals have overlapping, but in the upper and lower salinity ranges non-congruent tolerance curves (Neumann, 1960; Kangas and Skoog, 1978; Symanowski and Hildebrandt, 2010). Survival rates of brackish water animals and freshwater animals brought to very high or very low salinities were significantly different which led to the definition of two ecotypes of this snail species, a BW ecotype and a FW ecotype (Wiesenthal et al., 2018). It seems that animals of these ecotypes, although both able to rapidly acclimate to a wide range of different salinities (phenotypic plasticity) have subtle genetic differences affecting their osmotolerance.

Considering this background, it was interesting to investigate whether snails from the two ecotypes are able to adjust their gene transcription, protein expression or enzymatic activities of ion transporting ATPases in response to changes in environmental salinity.

Results from semi-quantitative RT-PCR analyses of transcript levels of the alpha-subunit of the Na^+^/K^+^-ATPase or the A subunit of the V-ATPase in whole body extracts of individual animals did not reveal any differences in FW and BW animals that had been maintained in their original media. A minor increase was detected in the transcript abundances in BW animals that were acclimated to very high salinity (28‰) or to a very low salinity (0.5‰), but no changes occurred upon transfer of FW animals from their original medium to high salinity (18‰) (Fig. 2). We conclude that stepwise salinity changes (Fig. 1) do not provoke a gene regulatory response in the genes encoding subunits of ion transporting ATPases or major changes in mRNA stability.

Using suitable antibody preparations in semi-quantitative western blot analyses of whole body protein extracts did not reveal any differences in the alpha-subunit of the Na^+^/K^+^-ATPase or the B and C subunits of the V-ATPase in animals from both ecotypes upon maintaining them in their original medium (Fig. 3). Transfer of BW animals to very high (28‰) or very low salinity (0.5‰) were without any effects on the protein levels. When FW animals were transferred to high salinity (18‰), we detected minor increases in the alpha-subunit of the Na^+^/K^+^-ATPase as well as in the C subunit of the V-ATPase. Since expression levels of individual subunits of ion transporting ATPases should reflect the amount of the holoenzymes and the B subunit of the V-ATPase did not show any significant increase in its expression level under these conditions, we are doubtful that the minor changes in the other proteins of up to 1.5 times the control levels are biologically meaningful.

This conclusion is underlined by the results of ATPase activity measurements that we performed using protein extracts of FW or BW snails reared in their original medium or being transferred in a stepwise manner to extreme salinities (Fig. 4), which did not show any significant differences. While the overall activity of the Na^+^/K^+^-ATPase was similar to what other researchers measured in other invertebrate species (Caruso-Neves et al., 1998; Leong and Manahan, 1997), the V-ATPase activity was virtually zero (Fig. 4B). We concluded that the V-ATPase does not play any significant role in the adjustments that animals undergo when environmental salinity changes but may have other functions like nutrient uptake in the intestines. Since our snails had not been fed during the period of stepwise transfer to altered salinities, there may have been no need for active V-ATPase in these animals. It is well known from other invertebrates that the V-ATPase activity is regulated by dissociation or association of the cytosolic V_1_ subunit complex and the membrane-bound V_0_ subunit complex (Huss et al., 2011). In our case the association may not have been occurred so that we could measure the protein content, but no enzymatic activity of the V-ATPase. The activity of the Na^+^/K^+^-ATPase did not show any difference between FW and BW animals in their original media and no significant changes upon transfer of animals to media with very high, high or low salinities (Fig. 4A). Although there is a small chance that we missed relevant changes in ATPase activity due to very heterogenous activities of the Na^+^/K^+^-ATPase in different tissues of the snails (c.f. Patrick et al., 2006; Lin et al., 2016) we are convinced that the most likely explanation for the lack in ATPase activities is that these animals do not change their ion transport activity under conditions of changing environmental salinity.

Substantial up- or downregulation of transport ATPase abundance and function may not be biologically meaningful in these mostly osmoconforming animals, because the animals tolerate volume fluctuations due to osmotic gradients between body fluids and external medium very well (Symanowski and Hildebrandt, 2010). The ion transport ATPase activity is then needed to slowly re-establish the original volume over several hours or days. This can be achieved based on the constitutive activity of ion pumps. This may be the reason why large immediate shifts in medium salinity result in high mortality rates while slow and stepwise changes in medium salinity are fairly well tolerated (Wiesenthal et al., 2018).

Acclimation to chronically altered medium salinity may, however, be associated with changes in integumental permeability to water and ions, e.g. by changing transcellular (aquaporin) or paracellular (tight junction claudin, c.f. Duffy et al., 2011) permeabilities in epithelia. These hypotheses remain to be tested.

## MATERIALS AND METHODS

### Animals and salinity transfer regimes

Adult FW snails were collected at the ‘Schmaler Luzin’ in northern Germany (53°18′01.4″N 13°25′54.4″E) along with stones and substrate. Adult BW snails were collected on the southern shore of the Baltic Sea near the island of Riems (54°11′00″N 13°21′10.2″E) and the island of Hiddensee (54°34′39.0″N 13°06′48.0″E). Snails were kept in large tanks (60×30×30 cm, LWH) at room temperature (20–22°C) with the collected stones and substrate for a few days until the transfer experiments began. In these storage tanks, all individuals were held at their natural salinity conditions (FW animals at 0.5‰, BW animals at 8‰) that were achieved by dissolving relevant amounts of ‘Tropic Marine Classic’ sea salt (Dr. Biener GmbH, Wartenberg, Germany; main constituents listed in Table 1) in deionized water. Salinity of the experimental media (water with a salinity of 0.5‰ had an osmolality of 30 mOsmol/kg H_2_O, whereas water with a salinity of 8‰ had an osmotic concentration of approximately 230 mOsmol/kg H_2_O) were controlled using a Vapro 5520 osmometer (Wescor Inc., Logan, UT, USA) or a CO301 conductivity meter (VWR International, Darmstadt, Germany). The mean pH value of the incubation media was approximately 8.0.

**Table 1.**
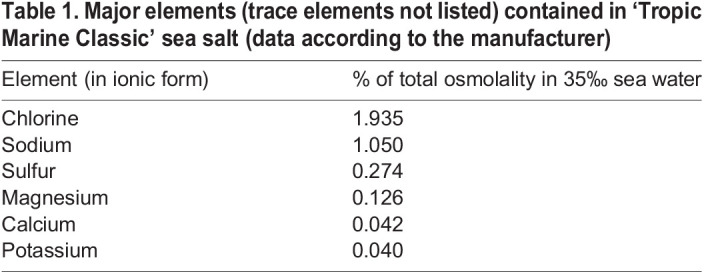
Major elements (trace elements not listed) contained in ‘Tropic Marine Classic’ sea salt (data according to the manufacturer)

The transfer experiments (Fig. 1) were generally carried out after adjusting the animals to the lab conditions for 2 to 14 days after the collection. Sexes were not separated for the experiments. For the duration of the transfer experiments, the snails were kept in small glass aquariums with 1 l of water (nine individuals each) and some pebbles for 10 days. The snails were kept under a 15:9 h light:dark regime in addition to the natural daylight cycle, ensuring at least 15 h of light a day. Water was changed during the transfers of animals to different salinities and survival was checked every 24 h. Animals were considered dead when they did not retract or show any sign of motion to the touch of a needle or if there was no resistance when trying to open the operculum. Exposure to the final salinities was limited to 5 days, because it had previously been shown that medium-induced changes in internal conditions of the snails were completed well within this period (Symanowski and Hildebrandt, 2010). The transfer regime of the experiment for FW individuals was composed of a control (maintenance at a salinity that matched their original medium) and high osmotic stress (up to 18‰). Individuals of the BW ecotype were treated similarly. Controls were kept in brackish water with a salinity of 8‰ (as in their original medium). Experimental BW animals experienced osmotic stress at the lower range (down to 0.5‰) or at a high salinity (up to 28‰). At the end of the transfer experiments, animals were stored at −20°C for 10 min for anaesthetization before the shells were removed. Wet weight of the remaining tissue was determined for each animal and tissue was stored in a 2 ml tube in liquid nitrogen until further use.

### Extraction of total RNA from animal tissues and semi-quantitative PCR

The RNA extraction was performed using TRIzol reagent (Thermo Fisher Scientific, Schwerte, Germany) according to the manufacturer's instructions. Briefly, 1 ml of TRIzol reagent was added to each sample and the tissue was homogenized (T8-ULTRA-TURRAX, IKA-Werke, Staufen, Germany). The homogenate was incubated for 5 min at room temperature to allow full solubilization of nucleic acids and proteins before 200 µl trichloromethane was added to each of the samples. The mixture was shaken vigorously for 15 s by hand and left for another 3 min at room temperature. Centrifugation for 15 min at 12,000 ***g*** and 4°C separated the samples in three different phases. The upper colorless aqueous phase contained the RNA and was transferred to fresh RNase-free tubes. To each sample 500 µl of 2-propanol was added and the content was mixed by inverting the tube. After 10 min of incubation at room temperature the samples were centrifuged for 10 min at 12,000 ***g*** and 4°C. The supernatant was discarded from the pelleted RNA at the bottom of the tube. The RNA was washed using 1 ml of 75% ethanol. Upon removal of the alcohol, residues were eliminated through evaporating for 15 min at 37°C. The dry RNA pellets were dissolved in RNase-free water by up and down pipetting. After a final incubation of the RNA solution at 57°C for 15 min, a 1:10 dilution was prepared, and RNA concentration and purity were measured photometrically. The integrity of the RNA was checked by agarose gel electrophoresis (2% gels) and ethidium bromide staining. The RNA was stored at −80°C until use.

Prior to cDNA synthesis, DNase I digestion was performed with each RNA isolate to eliminate any contaminating DNA. For each µg of RNA 1U RNase-free DNase I (EN0521, Thermo Fisher Scientific, Schwerte, Germany) was used. After 30 min at 37°C the reaction was stopped by adding 1 µl of a 50 mmol/l EDTA-solution per 10 µl of reaction volume followed by denaturation of the DNase at 65°C for 10 min.

Standard amounts of each RNA preparation were transcribed using the reverse transcriptase RevertAid (EP0441, Thermo Fisher Scientific, Schwerte, Germany). The 20 µl aliquots of reaction volume contained 300 ng total RNA, 1.5 µmol/l oligo(dT)_20_-primer and 200 U RevertAid. The reaction was performed following the RevertAid manufacturer's protocol.

Based on transcriptomic data (NCBI, accession number: SRR15300633), specific primers for the mRNAs of the Na^+^/K^+^-ATPase (alpha-subunit), the V-ATPase (subunit A) and beta-actin have been designed (Table 2). The primers were digitally tested for specificity and presence of potential self-complementary stretches using the NCBI primer blast function. PCR products were sequenced to verify the identity of the amplification products (Fig. S1).

**Table 2.**
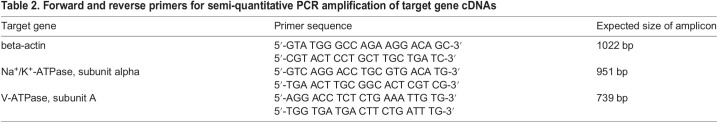
Forward and reverse primers for semi-quantitative PCR amplification of target gene cDNAs

All PCR reagents were obtained from New England Biolabs (Frankfurt/M., Germany). The 25 µl reaction volume of each PCR assay contained 2.5 µl 10x ThermoPol reaction buffer, 1 µl of a 10 mmol/l dNTP-mixture, 2.5 µl of a 10 µmol/l solution of forward primer, 2.5 µl of a 10 µmol/l solution of reverse primer, 2.5 µl cDNA, 0.5 µl *Taq* DNA Polymerase and 13.5 µl nuclease-free water. To avoid saturation, optimal cycle numbers were determined for each cDNA: 30 cycles were used for the Na^+^/K^+^-ATPase-cDNA and for the V-ATPase-cDNA, 25 or 20 cycles, respectively, were used for the cDNA of beta-actin.

The PCR products were separated in 2% agarose gels at 90 V. The cDNA bands in the gels were stained using ethidium bromide for 10 min before images were taken using the Gel Doc EZ Imager (Bio-Rad, Feldkirchen, Germany). Band intensities were measured by densitometry using Phoretix 1 D (Nonlinear Dynamics, Newcastle upon Tyne, UK). The relative band densities of Na^+^/K^+^-ATPase- or V-ATPase-related PCR products were normalized to those of β-actin in the same cDNA preparation and on the same gel. All experiments were performed in triplicate.

The whole procedure of semi-quantitative PCR was already successfully used in our group for the determination and quantification of temporal changes in gene expression both *in vitro* (endocrine disruptor-induced vitellogenin mRNA/cDNA abundances in isolated *Xenopus laevis* hepatocytes; Klann et al., 2004) and *in vivo* (feeding status-dependent hirudin mRNA/cDNA abundances in leech salivary gland cells; Lemke et al., 2016).

### Extraction of whole body proteins and western blot analyses

To obtain sufficiently high concentrations of proteins for the western blot experiments, two animals were pooled for each extraction. Each frozen snail tissue sample was homogenized 3x for 30 s on ice using a T8-Ultraturrax homogenizer (IKA-Werke, Staufen, Germany) in 10 µl of lysis buffer [100 mmol/l KCl, 20 mmol/l NaCl, 2 mmol/l MgCl_2_, 0.96 mmol/l NaH_2_PO_4_, 0.84 mmol/l CaCl_2_, 1 mmol/l EGTA, 0.5% (v/v) Tween 20, 25 mmol/l HEPES (free acid), pH 7.2] containing protease inhibitors (0.31 mmol/l aprotinin, 4.21 μmol/l leupeptin, 2.92 μmol/l pepstatin, 1 mmol/l PMSF and 0.33 mmol/l ortho-vanadate) per mg of tissue fresh weight. Homogenates were centrifuged at 16,200 ***g*** and 4°C to remove debris and protein concentrations were measured (Bradford, 1976) using BSA-solution as a standard. Aliquots were prepared for long term storage at −80°C in an equal volume of SDS sample buffer [50 mmol/l Tris (free base), 1% SDS, 0.2% bromophenol blue, 4% β-mercaptoethanol, 40% glycerol, pH 6.8].

After thawing the samples and brief heating to 95°C, 20 µg of total protein were separated in each lane of a 10% SDS-polyacrylamide gel in a mini-gel apparatus (Bio-Rad, Feldkirchen, Germany). Proteins were transferred to nitrocellulose membrane (NC 2, SERVA, Heidelberg, Germany) by wet blotting in buffer solution containing 48 mmol/l Tris (free base), 39 mmol/l glycine, 20% methanol (v/v), 1.3 mmol/l SDS, pH 9.2 at 100 V and 4°C.

Monoclonal antibodies against the alpha-subunit of Na^+^/K^+^-ATPase (1:500; DSHB, Iowa City, IA, USA) or beta-actin (1:10,000; Sigma-Aldrich, Taufkirchen, Germany) as well as a polyclonal antibody against the V1 subunit complex of the V-ATPase (1:3333; courtesy of Markus Huss, University of Osnabrück, Germany) were used as primary antibodies. Horse anti-mouse IgG-HRP or a goat anti-guinea pig IgG-HRP secondary antibodies (1:5000; Cell Signaling Technology, Frankfurt/M., Germany) were used in combination with a chemiluminescence substrate (Advansta Western Bright, Biozym, Hessisch Oldendorf, Germany) and the Intas Chemostar ECL imager (Intas, Göttingen, Germany). Band signal intensities were determined using Phoretix 1 D (Nonlinear Dynamics, Newcastle upon Tyne, UK). In order to correct for potential variations in exposure of different blots, the mean density of all bands on each blot image was used to normalize the densities of the individual bands. The Na^+^/K^+^-ATPase signals were normalized to the band intensities of beta-actin. The V-ATPase signals were normalized to the 45 kDa band in the Ponceau-stained blots.

### Extraction of proteins and measurements of ATPase activities

Sample extraction and activity assays were essentially performed as described previously (Lee and Watts, 1994; Tsai and Lin, 2007; Faleiros et al., 2010). Each frozen snail tissue sample was homogenized 3x for 30 s on ice using a T8-Ultraturrax homogenizer (IKA-Werke, Staufen, Germany) in 20 µl homogenization buffer (0.1% sodium deoxycholate, 250 mmol/l sucrose, 20 mmol/l imidazole, 6 mmol/l EDTA, 1 mmol PMSF, 14 µmol/l aprotinin, 1 µmol/l leupeptin, 1 µmol/l pepstatin, pH 6.8) per mg of tissue wet weight. To remove crude cellular debris, the homogenate was centrifuged at 1000 ***g*** for 15 min at 4°C. Each supernatant was transferred to a fresh tube and centrifuged again at 1000 ***g*** for 15 min at 4°C. Aliquots of the supernatants were prepared and immediately frozen in liquid nitrogen. For long term storage the samples were stored in −80°C. The protein concentration was measured as described above.

ATPase activities were measured photometrically using a pyruvate kinase/lactate dehydrogenase-linked assay. The consumption of ATP is monitored by measuring the loss of NADH (ε_340nm_=6220 l mol^−1^ cm^−1^) using an Infinite M200Pro microplate reader (Tecan, Crailsheim, Germany) in transparent 96-well plates (83.3924, Sarstedt, Numbrecht, Germany). The specific enzyme activity is determined by calculating the differences in the NADH decline rates between parallel assays in the absence of presence of specific inhibitors of the ATPases (Na^+^/K^+^-ATPase: 6 mmol/l ouabain or V-ATPase: 1 µmol/l bafilomycin A1).

The assay buffer used for Na^+^/K^+^-ATPase assays contained 50 mmol/l HEPES/Tris (pH 7.5), 40 mmol/l NaCl, 10 mmol/l KCl, 3 mmol/l MgCl_2_, 3.2 mmol/l phosphoenolpyruvate, 2 mmol/l ATP, 0.2 mmol/l NADH, 49 U/ml pyruvate kinase and 94 U/ml lactate dehydrogenase. The assay buffer used for V-ATPase assays contained 50 mmol/l HEPES/Tris (pH 7.5), 3 mmol/l KCl, 1.5 mmol/l MgCl_2_, 3.2 mmol/l phosphoenolpyruvate, 2 mmol/l ATP, 0.2 mmol/l NADH, 49 U/ml pyruvate kinase, 94 U/ml lactate dehydrogenase and 50 µmol/l of sodium orthovanadate to reduce the background activity corresponding to P-type ATPases.

Samples were thawed and the protein concentration was adjusted to 0.5 mg/ml using homogenization buffer. Assay buffer and homogenate were combined in a 96-well plate placed on ice. To start the reaction a mixture of ATP and NADH were added to a final volume of 200 µl. The microplate reader monitored the absorbance of NADH at 340 nm for at least 1.5 h at constant 25°C. Measurements were taken every 3 or 5 min, respectively, and the plate was shaken in between for 6 s. The slopes of the NADH absorbance changes became steady after approximately 30 min and were determined in the time interval between 60 and 70 min.

### Statistics

The measurements were tested for their normal distribution using the Kolmogorov–Smirnov test followed by the Levene's test to evaluate the homogeneity of variance. The mean values of the control groups were tested for statistical significance to the mean values of the corresponding experimental groups. If variances were equal between groups the two-sided *t*-test was used to test for significant differences of means, otherwise the Welch's *t*-test was used. Significant differences of means were presented as: *P*<0.05 (*), *P*<0.01 (**) or *P*<0.001 (***).

## Supplementary Material

Supplementary information
