# Supplementary material for: Expression levels and activities of energy-yielding ATPases in the oligohaline neritid snail Theodoxus fluviatilis under changing environmental salinities
Source: Biol Open. 2022 Feb 11;11(2):bio059190. doi: 10.1242/bio.059190 (PMC8844442; doi:10.1242/bio.059190)
Supplement: Supplementary information [file biolopen-11-059190-s1.pdf]

Biology Open (2022): doi:10.1242/bio.059190: Supplementary information

A

```
PCR_sample : CAACTGGTCGGCAAGGGCTCCCTGGCCGAGTCTGACAAGATCACTCTGGAAGTGGCCAAACTCATCAAAGACGACTACCTGCAGCAGAAC :  90
contig_6135: CAACTGGTCGGCAAGGGCTCCCTGGCCGAGTCTGACAAGATCACTCTGGAAGTGGCCAAACTCATCAAAGACGACTACCTGCAGCAGAAC :  90
contig_4285: CAACTGGTCGGCAAGGGCTCCCTGGCCGAGTCTGACAAGATCACTCTGGAAGTGGCCAAACTCATCAAGGACGACTACCTGCAGCAGAAC :  90
contig_4286: CAACTGGTCGGCAAGGGCTCCCTGGCCGAGTCTGACAAGATCACTCTGGAAGTGGCCAAACTCATCAAGGACGACTACCTGCAGCAGAAC :  90

PCR_sample : GGCTACACTCCCTATGACAGGTTCTGCCCCTTCTACAAGACTGTGGGCATGCTGAGGAACATAATCCACTTCTATGAGATGGCACGCAGC : 180
contig_6135: GGCTACACTCCCTATGACAGGTTCTGCCCCTTCTACAAGACTGTGGGCATGCTGAGGAACATAATCCACTTCTATGAGATGGCACGCAGC : 180
contig_4285: GGCTACACTCCCTATGACAGGTTCTGCCCCTTCTACAAGACTGTGGGCATGCTGAGGAACATCATCCACTTCTATGAGATGGCACGCAGC : 180
contig_4286: GGCTACACTCCCTATGACAGGTTCTGCCCCTTCTACAAGACTGTGGGCATGCTGAGGAACATCATCCACTTCTATGAGATGGCACGCAGC : 180

PCR_sample : GCCGTAGAAAAGACATCCCAGAGTGACCAGAAGATCACCTGGGCCATCATCCGTGACCAGCTCTCCAACACCATCTACAAGCTGTCCTCC : 270
contig_6135: GCCGTAGAAAAGACATCCCAGAGTGACCAGAAGATCACCTGGGCCATCATCCGTGACCAGCTCTCCAACACCATCTACAAGCTGTCCTCC : 270
contig_4285: GCCGTAGAAAAGACATCCCAGAGTGACCAGAAGATCACCTGGGCCATCATCCGTGACCAGCTCTCCAACACCATCTACAAGCTGTCCTCC : 270
contig_4286: GCCGTAGAAAAGACAGCCCAGAGTGACCAGAAGATCACCTGGGCCATCATCCGTGACCAGCTCTCCAACACCATCTATAAGCTGTCCTCC : 270

PCR_sample : ATGAAGTTCAAGGACCCAGTCAAGGATGGAGAGGCAAAGATCAAGGCAGACTTTGATGAGCTGTTCGAGGAGTTGAATGCTGGCTTCAGA : 360
contig6135 : ATGAAGTTCAAGGACCCAGTCAAGGATGGAGAGGCAAAGATCAAGGCAGACTTTGATGAGCTGTTCGAGGAGTTGAATGCTGGCTTCAGA : 360
contig4285 : ATGAAGTTCAAGGACCCAGTCAAGGATGGAGAGGCAAAGATCAAGGCAGACTTTGATGAGCTGTTCGAGGAGTTGAATGCTGGCTTCAGA : 360
contig4286 : ATGAAGTTCAAGGACCCAGTCAAGGATGGAGAGGCAAAGATCAAGGCAGACTTTGATGAGCTGTTCGAGGAGTTGAATGCTGGCTTCAGA : 360

PCR_sample : AATCTGGAGGACTAGACTGCTTGTCTGACTTGACCCTTCCCTGTGAAATAGCTCCCTGTCATCCCCTCTTCTCATCAGTGGACCAAGCAA : 450
contig_6135: AATCTGGAGGACTAGACTGCTTGTCTGACTTGACCCTTCCCTGTGAAATAGCTCCCTGTCATCCCCTCTTCTCATCAGTGGACCAAGCAA : 450
contig_4285: AATCTGGAGGACTAGACCGCTTGTCT-ACTTGACCCTTCCCTGTGAAATAGCTCCCTGTCATCCCCTCTTCTCATCAGTGGACCAAGCAA : 449
contig_4286: AATCTGGAGGACTAGACCGCTTGTCTGACTTGACCCTTTCCTGTGAAATAGCTCCCTGTCATCCC-TCTTCTCATCAGTGGACCAAGCAA : 449
                         TAG

PCR_sample : GAGACTTTTTCTCACTCTCCCATAACTGTCTAGATGCATAGCAGGTATCTAGAGCTGTGGCTAGTTTTGGGCAGATTGAATAGCTGCATC : 540
contig_6135: GAGACTTTTTCTCACTTTCCCATAACTGTCTAGATGCATAGCAGGTATCTAGAGCTGTGGCTAGTTTTGGGCAGATTGAATAGCTGCATC : 540
contig_4285: GAGACTTGTTCTCACTCTCCCATAACTGTCTAGATGCATAGCAGGTGTCTAGAGCTGTGGCTAGTTTTGGGCAGACTGAATAGCTGCATC : 539
contig_4286: GAGACTTGTTCTCACTCTCCCATAACTGTCTAGATGCATAGCAGGTGTCTAGAGCTGTGGCTAGTTTTGGGCAGACTGAATAGCTGCATC : 539

PCR_sample : AGTTTGTTGACATTGTTCTACCATTGACTAAACTTGTGTAGCATTATGAGCACCGGACAAATTTGTTGGGGTGTTTTCTGTGTAGAGCAG : 630
contig_6135: AGTTTGTTGACATTGTTCTACCATTGACTAAACTTGTGTAGCATTATGAGCACCGGACAAATTTGTTGGGGTGTTTTCTGTGTAGAGCAG : 630
contig_4285: AGTTTGTTGACATTGTTCTACCATTGACTGAACTTGTGTAGCATTATGAGCACCGGACAAATTTGTTGGGGTGTTTTCTGTGTAGAGCAG : 629
contig_4286: AGTTTGTTGACATTGTTCTACCATTGACTGAACTTGTGTAGCATTATGAGCACCGGACAAATTTGTTGGGGTGTTTTCTGTGTAGAGCAG : 629

PCR_sample : AAGTTTTTTCTCATCTTTGGGAGTTTCTTTTGC--GTATTGGTTTGATTTTTTCTTCATTTTTTAAATCCA : 699
contig_6135: AAGTTTTTTCTCATCTTTGGGAGTTTCTTTTGC--GTATTGGTTTGATTTTTTCTTCATTTTTTAAATCCA : 699
contig_4285: AAGTTTTTTCTAATCTTTGGGAGTTTCTTTTGC--GTATTGGTTTGATTTTTTCTTCATTTTTTAAATCCA : 698
contig_4286: AAGTTTTTTCTAATCTTTGGGAGTTTCTTTTGTATGTATTGGTTTGATTTTTTCTTCATTTTTTAAATCCA : 700
```

Biology Open • Supplementary information

B

```
PCR_sample1: GTCAGGACCTGCGTGACATGACCCCAGCCCAGATCGACGAGATCCTGAGGAACCACTCCGAGATTGTCTTTGCCCGCACCTCGCCCCAGC :   90
PCR_sample2: GTCAGGACCTGCGTGACATGACCCCGGCCCAGATCGACGAGATCCTGAGGAACCATTCCGAGATTGTCTTTGCCCGCACCTCGCCCCAGC :   90
contig_8808: GTCAGGACCTGCGTGACATGACCCCGGCCCAGATCGACGAGATCCTGAGGAACCACTCCGAGATTGTCTTTGCCCGCACCTCGCCCCAGC :   90
contig_8809: ------------------------------------------------------------------------------------------ :    -
contig_8810: ------------------------------------------------------------------------------------------ :    -
contig_9748: GTCAGGACCTGCGTGACATGACCCCGGCCCAGATCGACGAGATCCTGAGGAACCACTCCGAGATTGTCTTTGCCCGCACCTCGCCCCAGC :   90

PCR_sample1: AGAAGCTGATCATCGTGGAGGGATGCCAACGACAGGGCGCCATCGTGGCCGTGACTGGTGACGGTGTCAACGACTCGCCCGCCCTCAAGA :  180
PCR_sample2: AGAAGCTGATCATCGTGGAGGGATGCCAACGACAGGGCGCCATCGTGGCCGTGACTGGTGACGGTGTCAACGACTCGCCCGCCCTCAAGA :  180
contig_8808: AGAAGCTGATCATCGTGGAGGGATGCCAACGACAGGGCGCCATCGTGGCCGTGACTGGTGACGGTGTCAACGACTCGCCCGCCCTCAAGA :  180
contig_8809: ------------------------------------------------------------------------------------------ :    -
contig_8810: ------------------------------------------------------------------------------------------ :    -
contig_9748: AGAAGCTGATCATCGTGGAGGGATGCCAACGACAGGGAGCCATCGTGGCCGTGACTGGTGATGGTGTCAATGACTCGCCCGCCCTCAAGA :  180

PCR_sample1: AGGCTGATATCGGTGTTGCCATGGGTATTGCTGGCAGTGACGTCAGCAAACAGGCAGCTGACATGATCCTGCTGGACGACAACTTTGCCT :  270
PCR_sample2: AGGCTGATATCGGTGTTGCCATGGGAATTGCTGGCAGTGACGTCAGCAAACAGGCAGCTGACATGATCCTGCTGGACGACAACTTTGCCT :  270
contig_8808: AGGCTGATATCGGTGTTGCCATGGGAATTGCTGGCAGTGACGTCAGCAAACAGGCAGCTGACATGATCCTGCTGGACGACAACTTTGCCT :  270
contig_8809: --------------------------------------------------CAGGCAGCTGACATGATCTTGCTGGACGACAACTTTGCCT :   40
contig_8810: --------------------------------------------AGCAAACAGGCAGCTGACATGATCCTGCTGGACGACAACTTTGCCT :   46
contig_9748: AGGCTGATATCGGTGTTGCCATGGGAATTGCTGGCAGTGACGTCAGCAAACAGGCAGCTGACATGATCTTGCTGGACGACAACTTTGCCT :  270

PCR_sample1: CCATCGTCACTGGTGTTGAGGAAGGCCGTCTGATCTTCGACAACCTGAAGAAGTCCATCGCCTATACCCTCACCTCCAACATTCCTGAGA :  360
PCR_sample2: CCATCGTCACTGGTGTTGAGGAAGGCCGTCTGATCTTTGACAACCTGAAGAAGTCCATCGCCTATACCCTCACCTCCAACATTCCTGAGA :  360
contig_8808: CCATCGTCACTGGTGTTGAGGAAGGCCGTCTGATCTTTGACAACCTGAAGAAGTCCATCGCCTATACCCTCACCTCCAACATTCCTGAGA :  360
contig_8809: CCATCGTCACTGGTGTTGAGGAAGGCCGTCTGATCTTTGACAACCTGAAGAAGTCCATCGCCTATACCCTCACCTCCAACATTCCTGAGA :  130
contig_8810: CCATCGTCACTGGTGTTGAGGAAGGCCGTCTGATCTTCGACAACCTGAAGAAGTCCATCGCCTATACCCTCACCTCCAACATTCCTGAGA :  136
contig_9748: CCATCGTCACTGGTGTTGAGGAAGGCCGTCTGATCTTTGACAACCTGAAGAAGTCCATCGCCTACACCCTCACCTCCAACATTCCTGAGA :  360

PCR_sample1: TCTCCCCATTCCTGCTGTTCATTCTGGCTGACATTCCCCTGCCCCTGGGAACCGTCACCATCCTGTGCATTGACTTGGGAACCGACATGG :  450
PCR_sample2: TCTCCCCATTCCTGCTGTTCATTCTGGCTGACATTCCCCTGCCCCTGGGAACCATCACCATCCTGTGCATTGACTTGGGAACCGACATGG :  450
contig_8808: TCTCCCCATTCCTGCTGTTCATTCTGGCTGACATTCCCCTGCCCCTGGGAACCATCACCATCCTGTGCATTGACTTGGGAACCGACATGG :  450
contig_8809: TCTCCCCATTCCTGCTGTTCATTCTGGCTGACATTCCCCTGCCCCTGGGAACCATCACCATCCTGTGCATTGACTTGGGAACCGACATGG :  220
contig_8810: TCTCCCCATTCCTGCTGTTCATTCTGGCTGACATTCCCCTGCCCCTGGGAACCATCACCATCCTGTGCATTGACTTGGGAACCGACATGG :  226
contig_9748: TCTCCCCATTCCTGCTGTTCATTCTGGCTGACATTCCCCTGCCCCTGGG----------------------------------------- :  409

PCR_sample1: TGCCCGCCATCTCCCTTGCCTACGAGCAGGCTGAGAACGACATCATGAAGCGCCAGCCCCGTGACCCCTTCAAGGACAAGCTTGTGAACG :  540
PCR_sample2: TGCCCGCCATCTCCCTTGCCTACGAGCAGGCTGAGAACGACATCATGAAGCGCCAGCCCCGTGACCCCTTCAAGGACAAGCTTGTGAACG :  540
contig_8808: TGCCCGCCATCTCCCTTGCCTACGAGCAGGCTGAGAACGACATCATGAAGCGCCAGCCCCGTGACCCCTTCAAGGACAAGCTTGTGAACG :  540
contig_8809: TGCCCGCCATCTCCCTTGCCTACGAGCAGGCTGAGAACGACATCATGAAGCGCCAGCCCCGTGACCCCTTCAAGGACAAGCTTGTGAACG :  310
contig_8810: TGCCCGCCATCTCCCTTGCCTACGAGCAGGCTGAGAACGACATCATGAAGCGCCAGCCCCGTGACCCCTTCAAGGACAAGCTTGTGAACG :  316
contig_9748: ------------------------------------------------------------------------------------------ :    -

PCR_sample1: AGAGACTGATCTCCATGGCCTACGGACAGATTGGAATGATCCAGGCCTCCGCTGGCTTCTTCACCTACTTTGTCATCATGGCGGAGAACG :  630
PCR_sample2: AGAGACTGATCTCCATGGCCTACGGACAGATTGGAATGATCCAAGCCTCCGCTGGCTTCTTCACCTACTTTGTCATCATGGCGGAGAACG :  630
contig_8808: AGAGACTGATCTCCATGGCCTACGGACAGATTGGAATGATCCAAGCCTCCGCTGGCTTCTTCACCTACTTTGTCATCATGGCGGAGAACG :  630
contig_8809: AGAGACTGATCTCCATGGCCTACGGACAGATTGGAATGATCCAGGCCTCCGCTGGCTTCTTCACCTACTTTGTCATCATGGCGGAGAACG :  400
contig_8810: AGAGACTGATCTCCATGGCCTACGGACAGATAGGGATGATCCAAGCCTCCGCTGGCTTCTTCACCTACTTTGTCATCATGGCGGAGAACG :  406
contig_9748: ------------------------------------------------------------------------------------------ :    -

PCR_sample1: GCTTCTGGATTTCACGTCTGCTGGGCATCCGCGCCAAGTGGGATTCTCTGGGAATCAACGACCTCCATGACTCCTACGGCCAGGAATGGA :  720
PCR_sample2: GCTTCTGGATTTCACGTCTGCTGGGCATCCGCGCCAAGTGGGATTCTCTGGGAATCAACGACCTCCATGACTCCTACGGCCAGGAATGGA :  720
contig_8808: GCTTCTGGATTTCACGTCTGCTGGGCATCCGCGCCAAGTGGGATTCTCTGGGAATCAACGACCTCCATGACTCCTACGGCCAGGAATGGA :  720
contig_8809: GCTTCTGGATTTCACGTCTGCTGGGCATCCGCGCCAAGTGGGATTCTCTGGGAATCAACGACCTCCATGACTCCTACGGCCAGGAATGGA :  490
contig_8810: GTTTCTGGATTTCACGTCTGCTGGGCATCCGCGCCAAGTGGGATTCTCTGGGAATCAACGACCTCCATGACTCCTACGGCCAGGAATGGA :  496
contig_9748: ------------------------------------------------------------------------------------------ :    -

PCR_sample1: CCTACGCCCAGCGCAAGAAGCTAGAGTACACCTGCCACACCGCCTTCTTCGTGTCCATCGTGGTGGTGCAGTGGGCCGACTTGATCATCT :  810
PCR_sample2: CCTACGCCCAGCGCAAGAAGCTAGAGTACACCTGCCACACCGCCTTCTTCGTGTCCATCGTGGTGGTGCAGTGGGCCGACTTGATCATCT :  810
contig_8808: CCTACGCCCAGCGCAAGAAGCTAGAGTACACCTGCCACACCGCCTTCTTCGTGTCCATCGTGGTGGTGCAGTGGGCCGACTTGATCATCT :  810
contig_8809: CCTACGCCCAGCGCAAGAAGCTAGAGTACACCTGCCACACCGCCTTCTTCGTGTCCATCGTGGTGGTGCAGTGGGCCGACTTGATCATCT :  580
contig_8810: CCTACGCCCAGCGCAAGAAGCTGGAGTACACCTGCCACACCGCCTTCTTCGTGTCCATCGTGGTGGTGCAGTGGGCCGACTTGATCATCT :  586
contig_9748: ------------------------------------------------------------------------------------------ :    -

PCR_sample1: GCAAGACCCGCAGGCTTTCGCTCTTCCAGCAGGGCATGAAGAACCACCGCCTGACTTTCGCCCTGTTCTTTGAGACGGCACTGGCCGCCT :  900
PCR_sample2: GCAAGACTCGCAGGCTTTCGCTCTTCCAACAGGGCATGAAGAACCACCGCCTGACTTTCGCCCTGTTCTTTGAGACGGCACTGGCCGCCT :  900
contig_8808: GCAAGACTCGCAGGCTTTCGCTCTTCCAACAGGGCATGAAGAACCACCGCCTGACTTTCGCCCTGTTCTTTGAGACGGCACTGGCCGCCT :  900
contig_8809: GCAAGACCCGCAGGCTTTCGCTCTTCCAGCAGGGCATGAAGAACCACCGCCTGACTTTCGCCCTGTTCTTTGAGACGGCACTGGCCGCCT :  670
contig_8810: GCAAGACTCGCAGGCTTTCGCTCTTCCAGCAGGGCATGAAGAACCACCGCCTGACTTTCGCCCTGTTCTTTGAGACGGCACTGGCCGCCT :  676
contig_9748: ------------------------------------------------------------------------------------------ :    -

PCR_sample1: TCCTCTGCTACTGCCCTGGCCTGGATAAGGGGCTTCGCATGCAGTTCATCAGGCCTTCCTGGTGGTTCCCAGCATTCCCTTTCTCAATTG :  990
PCR_sample2: TCCTCTGCTACTGCCCTGGCCTGGATAAGGGGCTTCGCATGCAGTTCATCAGGCCTTCCTGGTGGTTCCCAGCATTCCCTTTCTCAATTG :  990
contig_8808: TCCTCTGCTACTGCCCTGGCCTGGATAAGGGGCTTCGCATGCAGTTCATCAGGCCTTCCTGGTGGTTCCCAGCATTCCCTTTCTCAATTG :  990
contig_8809: TCCTCTGCTACTGCCCTGGCCTGGATAAGGGGCTTCGCATGCAGTTCATCAGGCCTTCCTGGTGGTTCCCAGCATTCCCTTTCTCAATTG :  760
contig_8810: TCCTCTGCTACTGCCCTGGCCTGGATAAGGGGCTTCGCATGCAGTTCATCAGGCCTTCCTGGTGGTTCCCAGCATTCCCTTTCTCAATTG :  766
contig_9748: ------------------------------------------------------------------------------------------ :    -

PCR_sample1: CCATCTTCATCTACGACGAGTGCCGCAAGTTCA : 1023
PCR_sample2: CCATCTTTATCTACGACGAGTGCCGCAAGTTCA : 1023
contig_8808: CCATCTTTATCTACGACGAGTGCCGCAAGTTCA : 1023
contig_8809: CCATCTTCATCTACGACGAGTGCCGCAAGTTCA :  793
contig_8810: CCATCTTCATCTACGACGAGTGCCGCAAGTTCA :  799
contig_9748: --------------------------------- :    -
```

Biology Open • Supplementary information

Fig. S1. Comparison of sequenced PCR products with the *Theodoxus fluviatilis*-ESTs deposited in the NCBI database (accession # SRR15300633).
A – Alignment of the sequence of the PCR product with sequences of different contigs (4285, 4286 and 6135) in the NCBI database for the A-subunit of V-ATPase. The label 'TAG' marks the position of the stop codon indicating that the occurence of single nucleotide deletions were limited to the non-coding regions of the gene product.
B - Alignment of the sequences of two PCR products with sequences of different contigs (8808, 8809, 8810 and 9748) in the NCBI database for the alpha-subunit of Na^+/K^+-ATPase
The bases marked by a dark background are completely identical in all aligned sequences, the bases marked by a dark grey background are identical in 3 of 4 (A) or 4 of 5 (B) aligned sequences. Light grey backgrounds mark bases that are identical in less than 3 (A) or 4 (B) of the aligned sequences.

Biology Open • Supplementary information

Table S1.

Click here to download Table S1

Table S2.

Click here to download Table S2

Table S3.

Click here to download Table S3

Table S4.

Click here to download Table S4

Table S5.

Click here to download Table S5

Table S6.

Click here to download Table S6

Table S7.

Click here to download Table S7

Biology Open • Supplementary information
